# Expert consensus on the role of supplementation in obstetrics and gynecology using modified delphi method

**DOI:** 10.1007/s00404-023-07310-3

**Published:** 2023-12-28

**Authors:** Mohamed Yehia Soliman, Omima Abdel Fattah Idris, Mohamed Momtaz, Mohamed Ashraf Kortam, Mohamed Amr ELNoury, Hisham Ali Saleh, Ayman Abulnour, Ashraf Abo Ali, Mostafa Abbas, Omar M. Shaaban, Adel Shafik Salah El din, Hassan Gaafar, Yasser Orief, Michael Safwat

**Affiliations:** 1 Obstetrics and Gynecology, Ain Shams University, Cairo, Egypt; 2 Obstetrics and Gynecology, Al Kasr Al Aini, Cairo University, Cairo, Egypt; 3 Obstetrics and Gynecology, Alexandria University, Alexandria, Egypt; 4 El-Madina Fertility Centers, Alexandria, Egypt; 5 Obstetrics and Gynecology, Zagazig University, Zagazig, Egypt; 6 Obstetrics and Gynecology, Assiut University, Assiut, Egypt; 7 Medical Affairs Department, Eva Pharma for Pharmaceuticals and Medical Appliances, Cairo, Egypt

**Keywords:** Micronutrients, Supplementation, Pregnancy, Fertility, Delphi, Consensus

## Abstract

**Purpose:**

To reach a consensus among obstetrics and gynecology experts on the effects of micronutrient supplementation on fertility and pregnancy to aid clinicians in decision-making and create a unified approach to managing micronutrient deficiencies in women, by performing a modified Delphi study.

**Methods:**

A three-round modified Delphi process was conducted among a Delphi panel of 38 Egyptian experts to define recommendations regarding the role of supplementation on fertility and pregnancy in women of reproductive age. A literature review was performed and supporting evidence was graded to help guide the recommendations based on available evidence.

**Results:**

A total of 62 statements were developed for discussion and voting. Out of the 62 statements, 60 statements reached expert consensus. Statements were divided into two domains. The first domain discussed the role of supplementation in fertility: optimizing natural fertility, polycystic ovary syndrome (PCOS), in vitro fertilization/intracytoplasmic sperm injection (IVF/ICSI), unexplained infertility, and endometriosis, whereas the second domain was concerned with the role of supplementation in pregnancy during the prenatal, antenatal, and postnatal periods.

**Conclusion:**

In this work, a modified Delphi methodology was implemented to reach a consensus on the use of micronutrient supplementation in women of reproductive age. These recommendations can help clinicians in their practice, guide future research, and identify gaps in the market for the pharmaceutical industry. This clinical guidance can be extrapolated to similar communities.

## What does this study add to the clinical work

**Table:** 

In this work, a modified Delphi methodology was successfully used to reach a consensus among Egyptian experts on the use of micronutrient supplementation in women of reproductive age. This agreement can help clinicians in their practice, guiding future research and identifying gaps in the market for the pharmaceutical industry. This clinical guidance can be extrapolated to similar communities.	

## Introduction

Micronutrients are vitamins and minerals needed by the body in minute amounts for optimum functioning and overall well-being [1]. Deficiencies in these micronutrients have serious adverse effects on health that impacts individuals’ morbidity and mortality [2]. Female adolescents and women of reproductive age are more prone to micronutrient deficiencies due to increased demand [2, 3]. These deficiencies are aggravated when women embark on pregnancy, childbirth, and lactation journey when requirements are expected to increase [3]. The inability to adequately obtain some micronutrients during this sensitive period puts the woman and her fetus at high risk of developing complications that could extend to later stages of life [3].

A recent population-representative survey that assessed the levels of iron, zinc, and folate in women of reproductive age estimated two-thirds of non-pregnant women worldwide have micronutrient deficiencies [2]. Other studies have reported micronutrient deficiencies to varying levels in pregnant women [4, 5]. While iron and folic acid supplementation have long been recommended in pregnancy, other micronutrients are gaining attention as reflected in the WHO updated guidelines on antenatal nutrition [6]. A Cochrane systematic review that evaluated 20 studies looking into multiple micronutrient supplementation (MMS) in pregnancy concluded that replacing iron-folate supplements with MMS could be justified [3].

Given the above, however, there is an ongoing debate among physicians and healthcare providers on whether to recommend micronutrient supplementation to their female patients or not, which micronutrient, and how much. This debate has stemmed from the absence of definitive guidelines that recommend micronutrient supplementation in women in different stages of their lives to cater to their changing needs and the fact that there is no sufficient evidence to support the role of these nutrients on women’s health.

The aim of this work is to reach a consensus among obstetrics and gynecology experts on the effects of micronutrient supplementation on fertility and pregnancy to aid clinicians in decision-making and create a unified approach to managing micronutrient deficiencies in women, by performing a modified Delphi study.

## Methodology

**Fig. 1:**
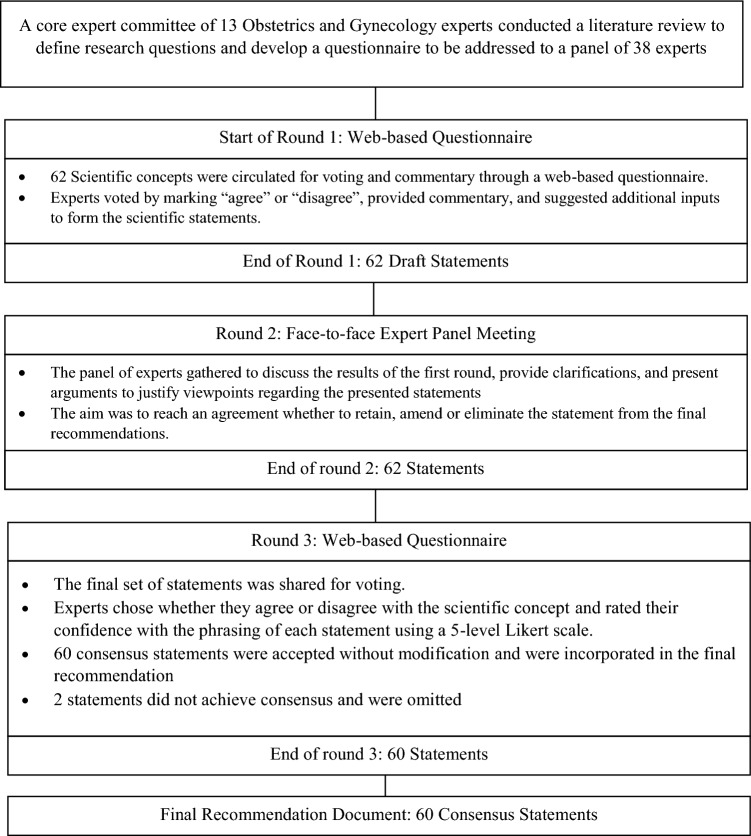
Modified Delphi process study design

A three-round modified Delphi Procedure (Fig. 1) was conducted between September 2022 and February 2023 to define recommendations regarding the role of supplementation in fertility and pregnancy in women of reproductive age. A core expert committee of 13 Obstetrics and Gynecology experts, representing the main academic entities from Egypt, convened to define research questions and develop a questionnaire to be addressed to a panel of experts. The panel of experts were chosen based on their expertise and relevant clinical practice within professional groups and scientific societies that directly influence patient care. They represented the main academic institutions and geographical regions all over Egypt.

The questions were divided into two main domains. The first domain focused on the role of supplements in fertility through five sub-categories (1) Optimizing natural fertility, (2) Polycystic ovary syndrome (PCOS), (3) In vitro fertilization/Intracytoplasmic sperm injection (IVF/ICSI), (4) Unexplained infertility and (5) Endometriosis. The second domain was concerned with the role of supplements in pregnancy and was also subdivided into three categories (6) The preconception period and during the first trimester, (7) The second and third trimesters, and (8) The postnatal period (The first 2 years after delivery).

A literature review was performed, and relative evidence was graded to help guide their recommendations based on available evidence.

In the first round of the Delphi process, a web-based questionnaire was conducted, including a clear explanation of the study objectives and instructions for participation. The questionnaire included open-ended questions to ensure the comprehensive inclusion of expert concepts for Delphi rounds 2 and 3.

Experts were asked to anonymously vote by marking “agree” or “disagree” with the scientific concept and were also requested to provide commentary and suggest additional inputs to form the scientific statements based on their real own practice. The first round of voting yielded a set of 62 scientific statements. Statements required at least 70% agreement from the panel as a cut-off to consider the statement in the final recommendations. Statements not meeting this cut-off will be modified according to the panel feedback and re-circulated for voting in round 2. In the second round, participating experts gathered in a face-to-face meeting to discuss the results of the first round, provide clarifications, and present arguments to justify viewpoints regarding the presented statements with the aim of reaching an agreement whether to retain, amend or eliminate the statement from the final recommendations.

In the third round, the final set of statements was anonymously shared for voting using the same method as round 1 to reduce dominance and group conformity. Experts were asked to choose whether they agree or disagree with the scientific concept as well as rating their confidence with the phrasing of each statement using a 5-level Likert scale.

Consensus was considered to have been reached if 70% or more of the experts showed agreement with the scientific statement in terms of concept and phrasing.

## Results

Thirty-eight experts were invited to participate in the development of this consensus, of whom 36 completed all three rounds. Following the three rounds of voting, data were analyzed, and consensus percentages were calculated.

Sixty-two statements were developed for discussion and voting. Forty-five statements discussed the effect of supplementation on fertility while 17 statements discussed the effect of supplementation during preconception, pregnancy, and the breastfeeding period. All statements reached consensus except two statements.

### Fertility

Expert consensus statements on the use of supplements in fertility are summarized in Table 1.

- 1. **Optimizing Natural Fertility**
  The participating experts recommended (agreement 94%) the use of supplements to optimize natural fertility due to their role in achieving general health and maintaining the homeostasis needed to promote natural pregnancy. Zinc, Vitamin C, Vitamin D, and Vitamin E have a potential role in promoting natural fertility although strong evidence is lacking. The use of folic acid is recommended (agreement 97.22%) to decrease the incidence of neural tube defects and optimize natural pregnancy. There is a consensus (agreement 94.44%) on the use of additional supplements to optimize natural fertility including inositol, omega 3, L-arginine, N-acetylcysteine, L-carnitine, selenium, lactoferrin, iron, calcium, iodine, and Co-enzyme Q10.
- 2. **Polycystic Ovary Syndrome (PCOS)**
  PCOS management involves diet and lifestyle modifications including supplementation. Experts recommended (agreement 97.22%) the use of some supplements as an integral part of the policy to approach PCOS cases, along with diet control and weight loss due to their role in reducing insulin resistance and improving ovulation. There is a consensus that the use of inositol is helpful in the management of PCOS-associated insulin resistance. The panel of experts advised the use of L-carnitine and acetyl L-carnitine for women with PCOS as they maintain cellular energy and reduce oxidative stress. They may also improve follicular development maturation and pregnancy outcomes.
- 3. **IVF/ICSI**
  There was a consensus among the panel of experts (agreement 100%) that the use of supplements in women undergoing IVF or ICSI treatments may be helpful, especially for women with PCOS and poor responders. Supplements such as inositol, L-carnitine, and acetyl L-carnitine could be recommended (agreement 97.22%) for women about to embark on their IVF/ICSI treatment journey. In cases of previous IVF/ICSI failure, the panel agreed that Co-enzyme Q10 supplementation may be used as it may improve mitochondrial function (agreement 88.89%).
- 4. **Unexplained Infertility**
  Experts agreed (agreement 94.44%) that the use of L-carnitine and Acetyl L-carnitine supplementation in cases of unexplained female infertility might be beneficial, as they have antioxidant potential that helps in oocyte development, supports ovum growth and maturity, and increases endometrial thickness and receptivity. There was also a consensus (83.33%) that co-enzyme Q10 has no known proven role to support its use in cases of unexplained infertility. However, it may improve ovarian function, increase oocyte number, support the ovarian response to stimulation, and improve implantation and pregnancy outcomes.
- 5. **Endometriosis**
  A consensus was reached (agreement 77.78%) on the use of supplements in women with endometriosis-associated pain due to the anti-inflammatory and antioxidant properties of supplements and their ability to reduce pain mediators.

**Table 1:** Experts’ Consensus Statements on the use of supplements in fertility

	Consensus level
1. *Optimizing Natural Fertility*	
1.1 The use of supplements to optimize natural fertility is recommended as they promote general health and maintain the homeostasis needed to promote natural pregnancy	94%
1.2 Vitamin C might be tried to promote natural fertility based on its antioxidant effect, although strong evidence is lacking	91.67%
1.3 Vitamin E is a strong antioxidant and might be tried as a natural supplement to improve natural fertility, although strong evidence is lacking	94%
1.4 Zinc is a strong antioxidant and might be tried as a natural supplement to improve natural fertility, although strong evidence is lacking	88.89%
1.5 Vitamin D supplementation may be of benefit in enhancing pregnancy rates in natural fertility in women with proven vitamin D deficiency	83.33%
1.6 The use of folic acid is recommended to decrease the incidence of neural tube defects and optimize natural pregnancy	97.22%
1.7 Additional supplements recommended to optimize natural fertility include inositol, omega 3, L-arginine, N-acetylcysteine, L-carnitine, selenium, lactoferrin, Tribulus herb, iron, calcium, iodine, and coenzyme Q10	94.44%
2. *Polycystic Ovary Syndrome (PCOS)*	
2.1 The use of some supplements should be an integral part of the policy to approach PCOS cases, hand in hand with diet control and weight loss, as they all culminate in reducing insulin resistance and improving ovulation	97.22%
2.2 The use of inositol is an emerging weapon to fight PCOS-associated insulin resistance with increasing scientific background day after day	97.22%
2.3 L-carnitine and acetyl L-carnitine may be advised for women with PCOS because they maintain cellular energy and reduce oxidative stress. They may also improve follicular development maturation and pregnancy outcomes	97.22%
2.4 L-arginine and N-acetyl cysteine supplementation may be beneficial in women with PCOS through improving uterine blood flow, which is proposed to increase nutrient and oxygen delivery to maturing oocytes with consequent enhancement of follicular growth, oocyte quality, and fertilization	94.44%
2.5 Clinical evidence is still lacking to support the role of coenzyme Q10 in women with PCOS. In spite of the presence of data, it can improve insulin sensitivity and mitochondrial function	97.22%
2.6 Vitamin C supplementation may be beneficial in women with PCOS as it is essential for the maintenance and synthesis of collagen, which is required for follicle growth, repair of the ovulated follicle, and corpus luteum development. It also has a potent antioxidant effect	75%
2.7 There is some evidence that Vitamin E may improve folliculogenesis and endometrial receptivity	88.89%
2.8 Zinc supplementation might be contemplated as a part of antioxidant therapy in cases of PCOS, especially due to its crucial role in insulin metabolism. Also, it affects ovarian development and oocyte maturation	86.11%
2.9 Selenium is a strong antioxidant and may have a role in the treatment of women with PCOS, but clinical evidence of its benefits is still lacking	91.67%
2.10 Vitamin D supplementation may have a role in the management of women with PCOS, who have proven vitamin D deficiency	86.11%
2.11 Vitamins B5, B6, B12, and folic acid could be recommended for women with PCOS because they regulate homocysteine levels and decrease insulin resistance, which is commonly elevated in women with PCOS	83.33%
2.12 Women suffering from PCOS may benefit from supplements with inositol, melatonin, Thioctic acid, L-carnitine, iron, calcium, chromium, omega-3, curcumin, and magnesium	86.11%
3. *IVF/ICSI*	
3.1 The use of supplements in women undergoing IVF/ICSI may be helpful, especially in PCOS and poor responder patients	100%
3.2 Inositol supplementation may be useful for women with PCOS receiving IVF/ICSI	91.67%
3.3 The use of L-carnitine and acetyl L-carnitine supplements could be recommended for women receiving IVF/ICSI, especially IVF PCOS patients	97.22%
3.4 The use of L-arginine and N-acetylcysteine supplements may enhance endometrial thickness and vascularity and may be prescribed for some women receiving IVF/ICSI, especially in cases of previous failure and in women with thin endometrium	94.44%
3.5 Co-enzyme Q10 supplementation may improve mitochondrial function and may be used in some women receiving IVF/ICSI, especially in cases of previous failure	88.89%
3.6 The use of vitamin C is of doubt to be beneficial in IVF/ICSI patients and its role needs to be re-elucidated	88.89%
3.7 Vitamin E is a strong antioxidant that may improve gametes quality and may be used in some cases of IVF/ICSI	88.89%
3.8 Zinc is a strong antioxidant that may improve gametes quality and may be used in some cases of IVF/ICSI with insulin resistance	83.33%
3.9 Selenium supplementation might be of benefit to IVF patients through its antioxidant effect. However, its role needs to be further evaluated	97.22%
3.10 Vitamin D supplementation is recommended for women receiving IVF/ICSI with proven vitamin D deficiency	88.89%
3.11 The use of folic acid is recommended to decrease the incidence of fetal neural tube defects in IVF patients	97.22%
3.12 No other supplements are known to be of proven value to be recommended to IVF patients	77.78%
4. *Unexplained Infertility*	
4.1 According to the available evidence, there is no known proven role to support the use of supplements in unexplained infertility. However, these supplements may reduce oxidative stress, improve oocyte maturation and endometrial quality	97.22%
4.2 There is no clear evidence that inositol can benefit women with unexplained infertility	97.22%
4.3 Supplementing L-carnitine and Acetyl L-carnitine in cases of unexplained female infertility might be beneficial, as they have antioxidant potential that helps in oocyte development, supports ovum growth and maturity, and increases endometrial thickness and receptivity	94.44%
4.4 According to the available evidence, there is no known proven role to support L-Arginine and N-Acetylcysteine supplementation in cases of unexplained infertility. However, they may play a role in folliculogenesis and improve the overall function of ovaries due to enhanced vascularity and reduced oxidative stress	91.67%
4.5 According to the available evidence, there is no known proven role to support the use of co-enzyme Q10 in cases of unexplained infertility. However, it may improve ovarian function, increase oocyte number, support the ovarian response to stimulation, and improve implantation and pregnancy outcomes	83.33%
4.6 Although evidence is lacking, vitamin C, being the most potent extracellular antioxidant may be beneficial in the management of unexplained infertility through improving implantation, supporting blood vessel integrity, improving ovulation, and reducing oxidative stress, all of which may decrease the time to a successful pregnancy	75%
4.7 Vitamin E is a strong antioxidant that could be used as an empirical treatment in unexplained infertility	88.89%
4.8 Zinc supplementation may be beneficial in cases of unexplained infertility as it regulates ovarian development, improves oocyte maturation and ovulation, has a crucial role in the synthesis, storage, secretion, function, and action of insulin and improves metabolic parameters	88.89%
4.9 According to the available evidence, there is no known proven role to support selenium supplementation in women with unexplained infertility as its levels are highly decreased in these cases. Its antioxidant capacity may improve insulin metabolism and lipid profile, boost immunity, and decrease time to pregnancy	91.67%
4.10 Vitamin D supplementation may be used in cases of unexplained infertility with proven vitamin D deficiency	94.44%
4.11 The use of folic acid is recommended to decrease the incidence of fetal neural tube defects in unexplained infertility patients	97.22%
4.12 No other supplements are known to be of proven value to be recommended in unexplained Infertility patients	88.89%
5. *Endometriosis*	
5.1 The use of supplements could be used to promote pregnancy in some women with endometriosis-associated infertility, but clinical evidence is lacking	86.11%
5.2 The use of supplements may be prescribed in women with endometriosis-associated pain due to the anti-inflammatory and antioxidant properties of dietary supplements and their ability to reduce pain mediators	77.78%

### Preconception, pregnancy, and postnatal breastfeeding

Expert consensus statements on the use of supplements in preconception, pregnancy, and postnatal breastfeeding period are listed in Table 2.

6. **Preconception Period and During the First Trimester of Pregnancy**
  The participating experts recommended MMS for women during the preconception period and the first trimester of pregnancy (agreement 88.89%), as they play a role in fetal programming, decreases the risk of anemia and hypertension, improve pregnancy outcomes, especially in high-risk pregnancies, and support mineralization. Among the supplements recommended for women in this vulnerable period, iron supplementation is recommended (agreement 97.22%) for anemic women in the preconception period and during the first trimester of pregnancy to improve anemia, enhance tissue oxygenation, reduce the risk of fetal morbidity and low birth weight, and improve the formation of blood elements. Another consensus was reached (agreement 97.22%) on the use of liposomal iron as an iron supplementation option for women in the preconception period and during the first trimester of pregnancy, as it may have better absorption and bioavailability profiles, lower side effects, and improved compliance. Omega-3 fatty acids supplementation is also recommended (agreement 83.33%) for women in the preconception period and during the first trimester of pregnancy, as it supports fetal development, can protect against pregnancy-induced hypertension, and in higher doses reduces the risk of preterm labor as the Egyptian diet is probably lacking.
7. **The second and third trimesters of pregnancy**
  The panel of experts recommended (agreement 97.22%) multiple micronutrient combinations, including iron and folic acid, as a superior option to iron and folic acid combination to aid in fetal development and support healthy pregnancy in accordance with recent WHO findings [3]. The combined supplements reduce adverse pregnancy outcomes, like low birth weight, small for gestational age births, and premature labor. Iron supplementation is recommended (agreement 100%) for women in the second and third trimesters of pregnancy, as it may have a role in the correction and prevention of iron deficiency anemia. Omega-3 supplementation is also recommended (agreement 86.11%) for women in the second and third trimesters of pregnancy because it may prevent preeclampsia and preterm labor and support fetal brain and eye development.
8. **The first 2 years after delivery**
  Following birth, experts recommended (agreement 91.67%) multiple micronutrients use for women during the first two years after delivery. At this stage, supplements improve milk secretion, support breastfed newborn growth, enhance the mother’s mental health, protect the mother from osteoporosis and fatigue, and enhance maternal body rejuvenation. Iron supplementation is recommended (agreement 94.44%) for women during the first two years after delivery to prevent and treat postpartum anemia. Also, it is essential for women with heavy menstruation, especially those using non-hormonal IUDs.

**Table 2:** Experts Consensus Statements on the use of supplements in Preconception, pregnancy, and postnatal breastfeeding

	Consensus Level
6. *Preconception period and during the first trimester of pregnancy*	
6.1 Supplements are recommended for women during the preconception period and the first pregnancy trimester, as many women may suffer from multiple concurrent micronutrient deficiencies during these periods. The use of supplements may improve anemia and reduce pregnancy complications, like abortion and preeclampsia	91.67%
6.2 Multiple micronutrient supplementation is recommended for women during the preconception period and the first trimester of pregnancy, as they play a role in fetal programming, decrease the risk of anemia and hypertension, improve pregnancy outcomes especially in high-risk pregnancies, and support mineralization	88.89%
6.3 Iron supplementation is recommended for anemic women in the preconception period and during the first trimester of pregnancy to improve anemia, enhance tissue oxygenation, reduce the risk of fetal morbidity and low birth weight, and improve the formation of blood elements	97.22%
6.4 The use of liposomal iron is rising nowadays as an iron supplementation option for women in the preconception period and during the first trimester of pregnancy, as it may have better absorption and bioavailability profiles, lower side effects, and improved compliance	97.22%
6.5 Omega-3 fatty acids supplementation is recommended for women in the preconception period and during the first trimester of pregnancy, as it supports fetal development, can protect against pregnancy-induced hypertension, and in higher doses reduces the risk of preterm labor	83.33%
7. *The second and third trimesters of pregnancy*	
7.1 Supplements are recommended to women in the second and third trimesters of pregnancy to support fetal development. Supplements at these stages can also decrease perinatal and maternal mortality and improve pregnancy outcomes	100%
7.2 Multiple micronutrient combination, including iron and folic acid, is recommended as a superior option to iron and folic acid combination to aid in fetal development and support a healthy pregnancy. The combined supplements reduce adverse pregnancy outcomes, like low birth weight, small for gestational age births, and premature labor	97.22%
7.3 There are multiple micronutrients needed in the second and third trimesters of pregnancy when nutritional adequacy is uncertain. These include zinc, calcium, magnesium, iron, vitamin B complex, omega-3, vitamin D, iodine, copper, vitamin A, vitamin C, vitamin D, vitamin E, beta carotene, calcium, selenium, and lactoferrin	97.22%
7.4 Iron supplementation is recommended to women in the second and third trimesters of pregnancy, as it may have a role in the correction and prevention of iron deficiency anemia. It may also improve pregnancy outcomes and decreases the risk of iron deficiency complications, especially obstetric hemorrhage	100%
7.5 Omega-3 supplementation is recommended for women in the second and third trimesters of pregnancy because it may prevent preeclampsia and preterm labor and support fetal brain and eye development	86.11%
7.6 Further supplementations are recommended when nutritional adequacy is uncertain during the second and third trimesters of pregnancy. They include calcium, iron, vitamin C, magnesium, iodine, vitamin B, lactoferrin, vitamin E, zinc, vitamin D, L-carnitine, and copper	100%
8. *The first 2 years after delivery*	
8.1 Multiple micronutrients are recommended for women during the first two years after delivery. At this stage, supplements improve milk secretion, support breastfed newborn growth, enhance the mother’s mental health, protect the mother from osteoporosis and fatigue, and enhance maternal body rejuvenation	91.67%
8.2 Multiple micronutrients are recommended for women during the first two years after delivery. This is because micronutrient supplementation needs to increase during lactation. Therefore, supplementation is warranted. Refer to Sect. 8.1 for their functional roles	91.67%
8.3 Iron supplementation is recommended for women during the first two years after delivery. This is to prevent and treat postpartum anemia. Also, it is essential for women with heavy menstruation, especially those using non-hormonal IUDs	94.44%
8.4 Further supplements that can be recommended for women during the first two years after delivery include magnesium, calcium, vitamins B, C, D, and E, zinc, beta carotene, biotin, and iodine	72.22%

There were two statements that didn’t reach a consensus summarized in Table 3.

**Table 3:** Statements that did not reach a consensus

Statement	Consensus Level
1. Selective additional supplements can be safely recommended during the preconception and the first trimester of pregnancy including folic acid or methyl folate, omega-6, vitamin D, vitamin B group, calcium, magnesium, zinc, and iodine	69.44%
2. Omega-3 supplementation is recommended for women during the first two years after delivery, as it may help in the neural development of the newborn and infants and benefit the mother’s physical and mental health	58.33%

## Discussion

### Optimizing natural fertility

This consensus appraised the role of supplementation in different stages and conditions in women’s lives.

Experts endorsed the use of supplements to optimize natural fertility. This recommendation matches the recent recommendations provided by the American Society for Reproductive Medicine (ASRM) and the Society for Reproductive Endocrinology and Infertility for optimizing natural fertility [7]. The recent committee opinion lists diet as one of the factors that practitioners should consider for optimizing pregnancy chances in women who desire to become pregnant and have no history of infertility [7]. The Nurses’ Health Study II evaluated the link between a specific diet, called the “Fertility diet”, and the risk of experiencing infertility due to an ovulatory disorder [8]. This study followed 17,544 women who had no history of infertility for 8 years along their journey to become pregnant [8]. Results demonstrated that women who followed the fertility diet, which was a healthy diet packed with multivitamins, had a lower risk of ovulatory disorder infertility [8]. Another study that assessed higher consumption of folic acid, vitamin B12, and vitamin D among a balanced diet of whole grains, dairy, soy foods, and seafood showed a higher probability of live birth in women undergoing Assisted Reproductive Technology (ART) [9]. Experts recommended the use of folic acid to decrease the incidence of neural tube defects. This goes in line with previous recommendations that have advised women trying to conceive to consume at least 400ug of folic acid daily to reduce the risk of neural tube defects [7]. Experts reached another consensus that advised women to consume Vitamin D in cases of deficiency. Other micronutrients were considered by the panel to promote natural fertility including Zinc, Vitamin C, and Vitamin E due to their antioxidant effects, although there is not enough evidence to support these recommendations.

Some studies have looked into the effect of individual micronutrients such as folic acid, omega 3, and multivitamins which appeared to have a possible positive impact on fertility [7]. Overall, although these recommendations may be beneficial to support natural fertility, there is a need for randomized controlled trials for more robust evidence.

### PCOS

Experts recommended the use of some supplements as an integral part of the policy to approach PCOS management in combination with diet control and weight loss. Among the supplements that could be helpful in PCOS management, Inositol is recently being explored as a treatment for PCOS-associated insulin resistance. The International evidence-based guideline for the assessment and management of polycystic ovary syndrome advised that Inositol (in any form) should be considered an experimental therapy in PCOS [10]. A systematic review highlighted the role of inositol as a safe and effective therapy option for PCOS. Further research is needed to confirm its role [11]. Consensus results showed that L-carnitine and acetyl L-carnitine may be advised in women with PCOS. Previous studies have documented its role when combined with ovulation induction drugs (Clomiphene Citrate and Letrozole)/metformin in improving ovulation, endometrial thickness, and pregnancy rate and improving insulin resistance respectively [12–14]. Another consensus was reached regarding the role of CoQ10 supplementation in women with PCOS although clinical evidence is lacking. Experts agreed that there is data to support its role in improving insulin sensitivity and mitochondrial function which may enhance PCOS symptoms. A randomized controlled trial evaluated the effect of coenzyme Q10 (CoQ10) and clomiphene citrate combination in clomiphene-citrate-resistant PCOS on ovulation induction [15]. Results showed that this combination improves ovulation and clinical pregnancy rates and is considered an effective and safe option early in the treatment before resorting to other alternatives [15]. Another study that looked into the role of CoQ10 and/or vitamin E on glucose homeostasis parameters and reproductive hormones in women with PCOS showed that CoQ10 supplementation had positive effects on serum fasting blood sugar and insulin levels [16]. It is also worth mentioning that experts agreed that selenium as a strong antioxidant may have a role in the treatment of women with PCOS although clinical evidence is lacking. Another randomized controlled trial on 70 women studied the effect of selenium supplementation on the metabolic profile of women with PCOS [17]. Results showed that supplementation with 200 ug selenium daily for 8 weeks among PCOS women improved insulin metabolism parameters, triglycerides, and VLDL-C levels [17]. Although there is no evidence to support the role of selenium in PCOS, selenium has an indirect effect on improving PCOS symptoms by enhancing insulin sensitivity and lipid profile [17] which goes in line with the experts’ recommendation.

### IVF/ICSI

Although the available evidence on the use of micronutrients to enhance fertility in couples undergoing IVF has increased, there is a lack of studies reporting the relationship between micronutrients and IVF outcomes [18]. A recent systematic review reported the positive impact of micronutrient supplementation on pregnancy outcomes of IVF therapy. [18] Conducting large clinical trials using micronutrient supplementation is necessary to evaluate their potential effects on pregnancy outcomes in patients undergoing IVF therapy. Experts agreed that the use of supplements in women undergoing IVF or ICSI treatments may be helpful for women about to start their IVF/ICSI journey or who had previous IVF/ICSI failure. They agreed that supplements such as inositol, L-carnitine, co-enzyme Q10, and acetyl L-carnitine could be recommended for this category of women. However, only low-quality evidence has supported the role of antioxidants in increasing live births [19].

### Unexplained fertility

The role of micronutrient supplementation to support fertility has been previously suggested [1]. Although few randomized controlled trials have been conducted, there is evidence to support the small but beneficial effects of multiple micronutrient supplementation on fertility including increased probability of becoming pregnant and a shorter time to achieve pregnancy [1]. A study that investigated Vitamin E effect on controlled ovarian stimulation in women with unexplained infertility concluded that Vitamin E supplementation may improve the endometrial response in women with unexplained infertility owing to its potential antioxidant and anticoagulant effects [20]. As a potent antioxidant, increased vitamin C in women with normal weight resulted in a shorter time to pregnancy as reported in a previous study [1]. Another review that evaluated the impact of periconceptional multiple-micronutrient supplementation on female fertility, discussed Vitamin D, zinc, and folic acid consumption among other nutrients [1]. Vitamin D deficiency in women has been potentially linked to a lower probability of becoming pregnant, whereas adequate levels of zinc and folate are necessary for the success of various stages in fertility such as oocyte fertilization and implantation, ovulation, and the menstrual cycle [1]. On the contrary, the ESHRE guidelines do not list multiple micronutrient supplementation as one of the alternative therapies used in the management of unexplained infertility. There was a consensus from the panel of experts on the potential use of Vitamin E, Vitamin C, Vitamin D, zinc, and folic acid for their probable benefits and added value in the management of unexplained fertility. Experts also agreed that the use of L-carnitine and Acetyl L-carnitine supplementation in cases of unexplained female infertility could be beneficial owing to their antioxidant effects. Although the role of co-enzyme Q10 is not fully understood in cases of unexplained infertility, it may increase oocyte number, support the ovarian response to stimulation, and pregnancy outcomes [21, 22]. Still, there is a need for large, prospective, randomized, rigorously controlled trials to provide robust evidence for the benefits of micronutrient supplementation on fertility in this group of women.

### Endometriosis

Endometriosis has been long linked to diet and nutrition [23–25]. Recent self-management approaches include dietary changes and lifestyle modifications [23–25]. While certain nutrient deficiencies can exacerbate symptoms of the disease [24], adequate intake of Vitamin D, Vitamin B, Vitamin C, Vitamin E, omega 3, zinc, and magnesium was suggested to improve endometriosis-associated pain [23–25]. Also, a recent study published in 2021 evaluated the effect of combined Vitamin C and Vitamin E Supplementation on Oxidative Stress Markers in Women with Endometriosis. Results showed that this combination has managed to significantly reduce malondialdehyde (MDA) and reactive oxygen species (ROS) which resulted in a reduction in the severity of dysmenorrhea, dyspareunia, and pelvic pain [26]. In line with these data, the panel of experts has recommended the potential use of dietary supplements in women with endometriosis-associated pain due to their anti-inflammatory and antioxidant properties. There was also a consensus on the possible use of supplements in women with endometriosis-associated infertility as they may promote pregnancy, although there is not enough evidence.

### Preconception, pregnancy, and the breastfeeding period

Experts recommended multiple micronutrient supplementation for women during the preconception period and first trimester (88.89%), second and third trimesters (97.22%), and the first two years after delivery (91.67%).

The participating experts agreed that iron and omega-3 fatty acids are recommended during the preconception period and in the first trimester. The recommendation of iron and omega-3 fatty acids supplementation continues in the second and third trimesters as highlighted in the consensus results. Multiple micronutrient combination, including iron and folic acid, is considered superior to iron and folic acid alone due to their role in fetal development and supporting healthy pregnancy. Further micronutrient supplementation is recommended in the second and third trimesters of pregnancy in case nutritional adequacy is uncertain. Additional micronutrients include zinc, calcium, magnesium, iron, vitamin B complex, omega-3, vitamin D, iodine, copper, vitamin A, vitamin C, vitamin D, vitamin E, beta carotene, calcium, selenium, and lactoferrin. For the first two years after delivery, iron supplementation received an agreement of 94.44% from the panel of experts due to its role in preventing and treating postpartum anemia. Also, it is essential for women with heavy menstruation, especially those using non-hormonal IUD.

In 2020, the World Health Organization (WHO) provided an updated recommendation to the previously published 2016 guidelines on the provision of multiple micronutrient supplements to pregnant women from populations with a high prevalence of maternal nutritional deficiencies [6, 27]. This recommendation serves to reduce pregnancy complications including the risks of low birth weight (LBW) and small for gestational age (SGA) compared to iron-folic acid supplementation alone [28]. Other studies have recommended the daily use of multiple micronutrient supplementation before conception and during pregnancy [29, 30]. These recommendations align with the consensus results as the experts agree on the need for multiple micronutrient supplementation in pregnant women in the preconception period and during pregnancy.

Due to increased iron demands during pregnancy, iron deficiency was the most common micronutrient deficiency reported in pregnant women [6]. Iron supplementation has long been recommended as part of the antenatal care routine in all pregnant women to avoid both maternal and fetal complications [6]. The WHO recommends daily elemental iron (30–60 mg) and folic acid (0.4 mg) to prevent maternal anemia [6]. A systematic review and meta-analysis has reported that prenatal iron maternal increases maternal hemoglobin, reduces iron deficiency, and reduces low birth weight [31]. These consensus results reflected similar recommendations that support iron supplementation in the preconception period and during pregnancy and go along to extend the recommendation for the next two years following delivery as a prophylaxis for postpartum anemia. The benefits of postpartum iron supplementation have been reported previously [32, 33].

Recent studies have reported an association between maternal micronutrient supplementation and child development [34, 35]. A randomized controlled trial found that preconception supplementation of multiple micronutrients and iron and folic acid (IFA) in pregnant women improved some aspects of intellectual functioning in their children at the age of 6–7 years compared with FA alone [34]. Another study has reported an association between prenatal micronutrient supplementation and improved language development in the first two years of life [35]. These studies support the conclusion that the benefits of maternal micronutrient supplementation go beyond the conception period and continue to have a positive impact on the offspring’s development later in life. A similar recommendation came from the expert panel that supports the use of multiple micronutrient combinations including iron and folic acid as a superior option to iron and folic acid alone to reduce adverse pregnancy outcomes, like low birth weight, small for gestational age births, and premature labor [36].

There was a consensus from the experts on the use of liposomal iron as an option for iron supplementation in pregnant women. Few studies have reported the added benefits of this novel technology as it offers better absorption, bioavailability, and tolerability profiles compared to available iron formulas, thus enhancing patient compliance [37, 38]

The panel of experts also recommended Omega-3 supplementation in the preconception period and during all three trimesters of pregnancy based on the benefits it provides. Available literature supports the potential role of omega-3 in reducing the risk of pre-term birth and low birth weight [39, 40]. Adequate DHA is believed to be necessary for fetal and early‐life brain development [40].

Overall, the consensus results go along with the current available evidence that supports the role of micronutrient supplementation in the development of safe and healthy pregnancy outcomes for both the mother and fetus.

## Conclusion

In this work, modified Delphi methodology was successfully used to reach a consensus among Egyptian experts on the use of micronutrient supplementation in women of reproductive age. This agreement can help clinicians in their practice, guiding future research and identifying gaps in the market for the pharmaceutical industry. This clinical guidance can be extrapolated to similar communities.

### Strengths/limitations

The use of Delphi methodology to develop this consensus is one of the study strengths that utilized expert opinions based on their clinical practice and the available evidence. Another strength is the expert panel that participated in the development of this consensus owing to their specialization and expertise. The geographical distribution of the expert panel from areas all around Egypt has also provided a wide range of opinions that better represents the country. This paper serves as guidance to streamline clinical practice in the absence of unified guidelines. Study limitations could include that not all participants participated in all Delphi rounds. Another limitation may be that all experts were from Egypt and having experts from other countries would have extended the recognition and adoption of this consensus among the region. Another point to consider is that even though this is an expert recommendation consensus, most statements were formulated based on Level 1 and Level 2 high-quality evidence.

### Future research

Future research should be directed towards conducting large-scale, double-blind, randomized controlled trials that evaluate the role of micronutrient supplementation in different stages and conditions of women’s life.
